# Stool pattern is associated with not only the prevalence of tumorigenic bacteria isolated from fecal matter but also plasma and fecal fatty acids in healthy Japanese adults

**DOI:** 10.1186/s12866-021-02255-6

**Published:** 2021-06-28

**Authors:** Daiki Watanabe, Haruka Murakami, Harumi Ohno, Kumpei Tanisawa, Kana Konishi, Kikue Todoroki-Mori, Yuta Tsunematsu, Michio Sato, Yuji Ogata, Noriyuki Miyoshi, Naoto Kubota, Jun Kunisawa, Keiji Wakabayashi, Tetsuya Kubota, Kenji Watanabe, Motohiko Miyachi

**Affiliations:** 1 Department of Physical Activity Research, National Institutes of Biomedical Innovation, Health and Nutrition (NIBIOHN), Tokyo, 162-8636 Japan; 2 Department of Clinical Nutrition, National Institutes of Biomedical Innovation, Health and Nutrition (NIBIOHN), Tokyo, 162-8636 Japan; 3 Department of Pharmaceutical Sciences, University of Shizuoka, Shizuoka, 422-8526 Japan; 4 School of Food and Nutritional Sciences, University of Shizuoka, Shizuoka, 422-8526 Japan; 5 Laboratory of Vaccine Materials, Center for Vaccine and Adjuvant Research, and Laboratory of Gut Environmental System, National Institutes of Biomedical Innovation, Health and Nutrition (NIBIOHN), Osaka, 567-0085 Japan; 6 Intestinal Microbiota Project, Kanagawa Institute of Industrial Science and Technology, Kanagawa, 243-0435 Japan; 7 Division of Diabetes and Metabolism, The Institute for Medical Science, Asahi Life Foundation, Tokyo, 103-0002 Japan; 8 Faculty of Sport Sciences, Waseda University, Saitama, 359-1192 Japan

**Keywords:** Tumorigenic bacteria, Stool pattern, Fatty acid, Cross-sectional study

## Abstract

**Background:**

Colibactin-producing *Escherichia coli* containing polyketide synthase (*pks*^+^ *E. coli*) has been shown to be involved in colorectal cancer (CRC) development through gut microbiota analysis in animal models. Stool status has been associated with potentially adverse gut microbiome profiles from fecal analysis in adults. We examined the association between stool patterns and the prevalence of *pks*^+^ *E. coli* isolated from microbiota in fecal samples of 224 healthy Japanese individuals.

**Results:**

Stool patterns were determined through factorial analysis using a previously validated questionnaire that included stool frequency, volume, color, shape, and odor. Factor scores were classified into tertiles. The prevalence of *pks*^+^ *E. coli* was determined by using specific primers for *pks*^+^ *E. coli* in fecal samples. Plasma and fecal fatty acids were measured via gas chromatography-mass spectrometry. The prevalence of *pks*^+^ *E. coli* was 26.8%. Three stool patterns identified by factorial analysis accounted for 70.1% of all patterns seen (factor 1: lower frequency, darker color, and harder shape; factor 2: higher volume and softer shape; and factor 3: darker color and stronger odor). Multivariable-adjusted odds ratios (95% confidence intervals) of the prevalence of *pks*^+^ *E. coli* for the highest versus the lowest third of the factor 1 score was 3.16 (1.38 to 7.24; *P* for trend = 0.006). This stool pattern exhibited a significant positive correlation with fecal isobutyrate, isovalerate, valerate, and hexanoate but showed a significant negative correlation with plasma eicosenoic acid and α-linoleic acid, as well as fecal propionate and succinate. No other stool patterns were significant.

**Conclusions:**

These results suggest that stool patterns may be useful in the evaluation of the presence of tumorigenic bacteria and fecal fatty acids through self-monitoring of stool status without the requirement for specialist technology or skill. Furthermore, it may provide valuable insight about effective strategies for the early discovery of CRC.

**Supplementary Information:**

The online version contains supplementary material available at 10.1186/s12866-021-02255-6.

## Background

Colorectal cancer (CRC) is the second most frequent cause of cancer death and the third most common cancer worldwide. Among all cancers, CRC contributes to a mortality rate of approximately 9% and an incidence of 10% [1]. The majority of disability-adjusted life years lost due to CRC primarily comes from years of life lost (95%), with years lived with disability only contributing 5% [2]. Reducing the number of CRC patients not only has a substantial impact on increased overall longevity for the human race but also has significant effects on reducing mean medical care costs. Thus, the discovery of simple targets that can lead to the early discovery of CRC risk is needed.

The fecal microbiome may contribute to CRC development because it shows changes in the very early stages [3]. Colibactin is a complex secondary metabolite produced by certain *Escherichia coli* strains in the gut that harbor the genomic island (*clb* gene cluster) encoding polyketide synthase (*pks*^+^ *E. coli*) [4–11]. Colibactin has been shown to cause genomic instability to mammalian cells by inducing DNA interstrand cross-links via DNA alkylation [7, 10, 11]. This phenomenon leads to DNA double-strand breaks [6–9] and cell cycle arrest [8]. Therefore, the presence of colibactin-producing *pks*^+^ *E. coli* in the gut microbiome may be a risk factor for CRC and may be a useful target for the identification of groups at high risk for both incidence and progression.

Previous studies have been reported that stool status variables such as stool shape [12–14], frequency [12, 15], and color [16] are associated with gut microbiome profiles from fecal analysis in healthy individuals and patients with acute gastroenteritis. Using a stool pattern approach, which considers a more comprehensive overview of the stool status, can provide more interpretable findings than studying single stool examinations because some stool status variables such as stool frequency and shape are related to each other [12].

We have previously reported that dietary intake was inversely related to the prevalence of *pks*^+^ *E. coli* in Japanese adults [17]. Indeed, dietary intervention has been reported to improve stool status variables, including stool frequency and shape with simultaneous increase in both fecal [18] and plasma [19] fatty acids. Therefore, to further clarify the association between stool status and the prevalence of *pks*^+^ *E. coli*, the association between stool status and fecal and plasma fatty acids need to be better understood. To our knowledge, these relationships have not been studied. In this study, we aimed: 1) to evaluate the association between stool patterns and prevalence of *pks*^+^ *E. coli* isolated from fecal samples, and 2) to investigate the relationship between stool patterns and plasma and fecal fatty acids in healthy Japanese individuals. We hypothesized that stool pattern variables were associated with the prevalence of *pks*^+^ *E. coli* and the levels of plasma and fecal fatty acids because stool patterns may reflect the gut microbiota.

## Results

### Participant characteristics

**Fig. 1:**
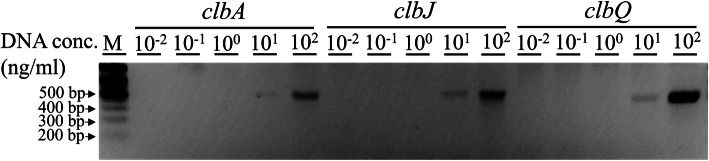
PCR products amplified at five concentrations (10^− 2^ to 10^2^ ng/mL) using *clb* genomic DNA purified from a colibactin-producing *E.coli*-50. The *clb* genes and the expected sizes of their amplicons in PCR are as follows: *clbA*, 613 bp; clbJ, 544 bp; and *clbQ*, 430 bp. M, DNA marker; conc., concentration

Table 1 shows the characteristics of participants in the analysis cohort. Of the 224 participants included, 60 participants were positive for *pks*^+^ *E. coli* isolated from fecal samples (26.8%). In the comparison between participants who were positive and negative for *pks*^+^ *E. coli*, the presence of *pks*^+^ *E. coli* tended to be lower in women but greater in alcohol drinkers. Other variables were not significantly associated. The minimum detection level of *clb* genes by polymerase chain reaction (PCR) was estimated at a concentration of 10 ng/mL as a DNA template (Fig. 1).

**Table 1:** Baseline characteristics of participants with or without *pks*^+^ *E. coli*

	*Total* (*n* = 224)	*pks*^+^ *E. coli* (*n* = 60)	*pks*^−^ *E. coli* (*n* = 164)	*p-*value
Age [years]^a^	58.7 (12.5)	57.2 (13.1)	59.2 (12.2)	0.290
Women [*n* (%)]^b^	165 (73.7)	37 (61.7)	128 (78.0)	**0.014**
Body mass index [kg/m^2^]^a^	22.4 (2.7)	22.4 (2.6)	22.4 (2.8)	0.859
Current smoking [*n* (%)]^b^	8 (3.6)	4 (6.7)	4 (2.4)	0.260
Alcohol drinker [*n* (%)]^b^	116 (51.8)	37 (61.7)	71 (43.3)	**0.015**
GTC everyday [*n* (%)]^b^	169 (75.4)	41 (68.3)	128 (78.0)	0.135
Hypertension [*n* (%)]^b^	39 (17.4)	12 (20.0)	27 (16.5)	0.537
Hyperlipidemia [*n* (%)]^b^	52 (23.2)	12 (20.0)	40 (24.4)	0.491
FH of cancer [*n* (%)]^b^	127 (56.7)	29 (48.3)	98 (59.8)	0.127
Antibiotics use [*n* (%)]^b^	26 (11.7)	8 (13.3)	18 (11.1)	0.648
Probiotics use [*n* (%)]^b^	28 (12.6)	6 (10.0)	22 (13.6)	0.476
Step counts [step/day]^a^	9610 (3241)	9582 (2887)	9610 (3374)	0.997
Employment [*n* (%)]^b^	141 (62.9)	43 (71.7)	98 (59.8)	0.102
Sleep time [min/day]^a^	398 (60)	405 (53)	395 (62)	0.251
Hemoglobin [g/dL]^a^	13.4 (1.1)	13.4 (1.3)	13.4 (1.1)	0.701
AST [IU/L]^a^	22.9 (6.9)	22.3 (6.4)	23.1 (7.1)	0.442
ALT [IU/L]^a^	18.3 (10.2)	18.0 (8.5)	18.4 (10.8)	0.794
γ-GTP [IU/L]^a^	29.3 (28.6)	28.7 (25.1)	29.5 (29.9)	0.851
FPG [mg/dL]^a^	87.0 (8.7)	87.7 (9.3)	86.8 (8.4)	0.489
FSI [μU/mL]^a^	4.0 (3.9)	3.6 (1.8)	4.1 (4.5)	0.414
HbA1c [%]^a^	5.5 (0.3)	5.5 (0.4)	5.5 (0.3)	0.527
Triglyceride [mg/dL]^a^	88.7 (57.4)	81.5 (46.2)	91.3 (60.9)	0.259
HDL-C [mg/dL]^a^	68.7 (18.6)	67.7 (21.9)	69.1 (17.3)	0.596
LDL-C [mg/dL]^a^	127 (30)	125 (29)	128 (30)	0.449

*ALT* alanine transaminase, *AST* aspartate transaminase, *FH* family history, *FPG* fasting plasma glucose, *FSI* fasting serum insulin, *GTC* green tea consumption, *HbA1c* hemoglobin A1c, *HDL-C* high-density lipoprotein-cholesterol, *LDL-C* low-density lipoprotein-cholesterol, *SD* standard deviation, *γ-GTP* γ-glutamyl transpeptidase. The *p*-values shown in bold are statistically significant (*p* < 0.05).

^a^Continuous variables are shown as mean with standard deviation and were analyzed by unpaired t-test. Body mass index was calculated as body weight (kg) divided by height squared (m^2^)

^b^Categorical variables are shown as number of individuals (%) and were analyzed using a *Χ*^*2*^ test

### Reproducibility of self-reported stool status

We evaluated the reproducibility of self-reported stool status variables, including stool volume, shape, color, and odor (Table 2). The weighted κ statistics (95% confidence interval [CI]) were 0.96 (0.93 to 0.99) for stool volume, 0.95 (0.92 to 0.98) for stool color, 0.97 (0.95 to 0.99) for stool shape, and 0.92 (0.86 to 0.98) for stool odor. Stool status that was self-reported twice was highly reproducible.

**Table 2:** Reproducibility of stool status assessed by self-reported questionnaire

	Agreement		Adjacent agreement		Disagreement		Weighted κ statistic	95% CI
	*n*	(%)	*n*	(%)	*n*	(%)		
Stool volume	120	(53.6)	46	(20.5)	17	(7.6)	0.96	(0.93 to 0.99)
Stool color	133	(59.4)	67	(29.9)	7	(3.1)	0.95	(0.92 to 0.98)
Stool shape	161	(71.9)	43	(19.2)	4	(1.8)	0.97	(0.95 to 0.99)
Stool odor	159	(71.0)	62	(27.7)	3	(1.3)	0.92	(0.86 to 0.98)

All values are shown as numbers of people and percentages and weighted κ statistics with 95% confidence interval (CI). The stool status was evaluated by each category as follows: stool volume [1 point (smaller) to 8 points (larger)], stool color [1 point (yellow) to 6 points (dark)], stool shape [1 (severe constipation; separate hard lumps) to 7 (severe diarrhea; liquid consistency with no solid pieces)], and stool odor [1 (no smell) to 4 (severe smell)]. Disagreement was defined as a difference of more than three categories between each variable. The weighted κ statistic can range from 0.00 (perfect disagreement) to 1.00 (perfect agreement)

### Multivariate analysis for stool patterns and *pks*^+^*E. coli* carriers

Table 3 shows the stool patterns identified by factorial analysis in this population. Three stool patterns accounted for 70.1% of all patterns seen (factor 1: lower frequency, darker color, and harder shape; factor 2: higher volume and softer shape; and factor 3: darker color and stronger odor).

**Table 3:** Stool patterns identified by factorial analysis using the 5-stool status

	Factor loadings		
	1	2	3
Stool status			
Stool frequency	**−0.817**	0.004	0.043
Stool volume	0.006	**0.895**	−0.035
Stool color	**0.534**	0.250	**0.450**
Stool shape	**−0.607**	**0.475**	0.012
Stool odor	−0.042	− 0.086	**0.931**
Variance explained by the factor (%)	26.7	21.9	21.5
Cumulative variance (%)	26.7	48.6	70.1

The values are expressed as factor load with the varimax rotation. Bolded values are stable factor load scores (≥0.4). The stool status was evaluated by each category as follows: stool frequency [1 point (2 or less times per week) to 6 points (7 or more times per week)], stool volume [1 point (smaller) to 8 points (larger)], stool color [1 point (yellow) to 6 points (dark)], stool shape [1 (severe constipation; separate hard lumps) to 7 (severe diarrhea; liquid consistency with no solid pieces)], and stool odor [1 (no smell) to 4 (severe smell)]

We evaluated the relationship between the prevalence of *pks*^+^ *E. coli* and stool patterns by multivariate logistic analysis (Table 4). The multivariable-adjusted odds ratios (95% CI) of the prevalence of *pks*^+^ *E. coli* for the highest versus the lowest third of the factor 1 score was significant at 3.16 (1.38 to 7.24; *P* for trend = 0.006); however, no significance was observed for any other stool pattern. In addition, stool status such as stool color and shape was significantly associated with the prevalence of *pks*^+^ *E. coli* (Supplementary Table 1).

**Table 4:** Odds ratios for stool patterns and prevalence of *pks*^+^ *E. coli* carriers

Stool patterns	Number or ORs (95% CI)						*p* for trend ^a^
	T1		T2		T3		
**Factor 1**							
*n*	77		71		76		
Case [*n* (%)]	14	(18.2)	16	(22.5)	30	(39.5)	
Model 1 ^b^	1.00	(Ref)	1.42	(0.61 to 3.27)	3.62	(1.63 to 8.03)	**0.002**
Model 2 ^c^	1.00	(Ref)	1.12	(0.47 to 2.67)	3.16	(1.38 to 7.24)	**0.006**
**Factor 2**							
*n*	75		74		75		
Case [*n* (%)]	17	(22.7)	22	(29.7)	21	(28.0)	
Model 1 ^b^	1.00	(Ref)	1.42	(0.67 to 2.98)	1.12	(0.52 to 2.40)	0.673
Model 2 ^c^	1.00	(Ref)	1.46	(0.67 to 3.18)	1.09	(0.49 to 2.44)	0.767
**Factor 3**							
*n*	75		75		74		
Case [*n* (%)]	18	(24.0)	22	(29.3)	20	(27.0)	
Model 1 ^b^	1.00	(Ref)	1.10	(0.51 to 2.35)	1.05	(0.49 to 2.23)	0.403
Model 2 ^c^	1.00	(Ref)	1.18	(0.53 to 2.62)	1.12	(0.50 to 2.51)	0.301

*Ref* reference. The prevalence rates of *pks*^+^ *E. coli* are shown as numbers of people and percentages. The detail of the three stool patterns are as follows. Factor 1: lower frequency, darker color, and harder shape; factor 2: higher volume and softer shape; and factor 3: darker color and stronger odor

^a^Statistical analysis was carried out using the likelihood ratio test for multivariate logistic analysis, and the odds ratios (ORs) and 95% confidence intervals (CI) were estimated. Bold *p* values are statistically significant (*p* < 0.05)

^b^Model 1 was adjusted for age (continuous) and sex (male or female)

^c^Model 2 was as model 1 plus mutual adjustment for body mass index (continuous), family history of cancer (yes or no), smoking status (never smoker, past smoker, or current smoker), step counts (continuous), alcohol drinker (yes or no), and green tea consumption (continuous)

### Correlation between stool status and plasma and fecal fatty acids

Table 5 shows the association of stool patterns with fatty acids derived from plasma and fecal samples. The factor 1 score was significantly positively correlated with fecal isobutyrate, isovalerate, valerate, and hexanoate but was significantly negatively correlated with plasma eicosenoic acid and α-linoleic acid, as well as fecal propionate and succinate. Other stool patterns showed no significant correlation. The correlations of stool status with plasma and fecal fatty acids, food and beverage consumption, and nutrients intake are tabulated and presented in Supplementary Tables 2, 3, 4 and 5, respectively.

**Table 5:** Correlation between stool patterns and plasma and fecal fatty acids

	Structure	*n*	Unit	Median	Interquartile range	Stool status pattern			
						1		2	3
**Plasma**									
Lauric acid	C12:0	221	μg/ml	1.8	(1.2 to 2.7)	0.02		−0.05	−0.05
Myristic acid	C14:0	223	μg/ml	21.4	(16.4 to 28.8)	−0.09		0.03	− 0.07
Palmitic acid	C16:0	223	μg/ml	668.7	(594.6 to 749.7)	−0.07		0.06	−0.04
Stearic acid	C18:0	223	μg/ml	227.3	(203.3 to 259.3)	−0.07		0.07	−0.03
Arachidic acid	C20:0	223	μg/ml	8.9	(7.8 to 10.0)	−0.11		− 0.06	− 0.05
Behenic acid	C22:0	223	μg/ml	21.7	(18.9 to 24.9)	0.00		−0.02	− 0.03
Lignoceric acid	C24:0	223	μg/ml	19.4	(0.8 to 22.8)	0.03		−0.02	−0.02
Total saturated fatty acids		223	μg/ml	969.9	(862.4 to 1092.5)	−0.07		0.06	−0.04
Oleic acid	C18:1n-9	223	μg/ml	568.6	(485.6 to 659.0)	−0.11		0.10	−0.01
Eicosenoic acid	C20:1n-9	223	μg/ml	4.2	(3.5 to 5.3)	−0.16	*	0.06	0.00
5,8,11-Eicosatrienoic acid	C20:3n-9	217	μg/ml	2.4	(1.5 to 3.2)	−0.05		−0.03	0.07
Erucic acid	C22:1n-9	48	μg/ml	1.2	(1.1 to 1.3)	−0.04		−0.04	− 0.12
Nervonic acid	C24:1n-9	223	μg/ml	36.1	(1.6 to 42.2)	−0.03		0.04	−0.10
Total n-9 fatty acids		223	μg/ml	610.7	(514.5 to 701.9)	−0.12		0.10	−0.01
Palmitoleic acid	C16:1n-7	223	μg/ml	48.7	(38.1 to 60.7)	−0.10		0.04	−0.08
Linoleic acid	C18:2n-6	223	μg/ml	956.8	(846.0 to 1111.3)	−0.02		0.05	−0.02
γ-linolenic acid	C18:3n-6	223	μg/ml	8.9	(5.8 to 12.9)	−0.06		−0.03	0.04
Eicosadienoic acid	C20:2n-6	223	μg/ml	7.2	(6.1 to 8.4)	−0.10		0.01	0.02
Dihomo-gamm-linolenic acid	C20:3n-6	223	μg/ml	38.7	(29.8 to 46.1)	−0.07		−0.05	0.05
Arachidonic acid	C20:4n-6	223	μg/ml	214.8	(184.5 to 249.7)	0.03		0.05	−0.01
Docosatetraenoic acid	C22:4n-6	223	μg/ml	4.8	(3.9 to 5.8)	0.04		0.03	0.06
Total n-6 fatty acids		223	μg/ml	1234.0	(1104.4 to 1389.5)	−0.01		0.03	−0.01
Myristoleic acid	C14:1n-5	221	μg/ml	1.2	(0.8 to 1.8)	−0.08		0.02	−0.10
α-linoleic acid	C18:3n-3	223	μg/ml	22.3	(16.7 to 30.0)	−0.14	*	0.03	−0.08
Eicosapentaenoic acid	C20:5n-3	223	μg/ml	65.9	(43.5 to 98.6)	−0.12		− 0.01	−0.12
Docosapentaenoic acid	C22:5n-3	223	μg/ml	16.5	(0.8 to 21.7)	−0.03		0.02	−0.05
Docosahexaenoic acid	C22:6n-3	223	μg/ml	137.4	(6.4 to 173.7)	−0.04		0.03	−0.10
Total n-3 fatty acids		223	μg/ml	236.4	(152.2 to 301.9)	−0.10		0.03	−0.11
Mono fatty acids		223	μg/ml	660.3	(560.4 to 756.6)	−0.12		0.10	−0.03
Polyunsaturated fatty acid		223	μg/ml	1482.0	(1292.1 to 1667.6)	−0.05		0.05	−0.07
n-3/n-6 fatty acids		223		0.18	(0.13 to 0.24)	−0.10		− 0.01	−0.12
Total fatty acids		223	μg/ml	3093.9	(2749.6 to 3512.7)	−0.07		0.07	−0.05
**Fecal**									
Formate	C1:0	58	mol/g	2.66	(0.83 to 4.59)	0.17		−0.10	−0.07
Acetate	C2:0	161	mol/g	57.41	(44.66 to 67.12)	−0.06		0.09	0.06
Propionate	C3:0	161	mol/g	16.40	(11.59 to 23.96)	−0.30	**	0.15	0.00
Lactate	C3:0	19	mol/g	0.08	(0.03 to 0.30)	0.04		−0.08	0.44
Isobutyrate	C4:0	161	mol/g	1.57	(0.95 to 2.19)	0.21	**	−0.11	−0.15
Butyrate	C4:0	161	mol/g	10.30	(6.59 to 17.52)	−0.07		0.06	−0.01
Succinate	C4:0	143	mol/g	0.18	(0.09 to 0.34)	−0.21	**	0.05	0.09
Isovalerate	C5:0	161	mol/g	1.01	(0.57 to 1.50)	0.27	**	−0.12	−0.14
Valerate (Pentanoate)	C5:0	160	mol/g	1.36	(0.66 to 2.06)	0.15	*	−0.03	−0.08
Hexanoate	C6:0	161	mol/g	0.06	(0.02 to 0.28)	0.32	**	−0.07	0.06
Total SCFA		161	mol/g	97.02	(72.12 to 116.97)	−0.13		0.11	0.03

Statistical analysis was performed by Spearman’s correlation analysis. The statistical significance *p* value is indicated as follows: if *p* < 0.05, single asterisk (*); if *p* < 0.01 double asterisk (**). If the results presented a positive correlation, the participants with higher adherence to each stool patterns mean to relate higher plasma and fecal fatty acids (conversely, a negative correlation indicates that they mean lower it)

## Discussion

In this study, we investigated the relationship between the prevalence of *pks*^+^ *E. coli* and stool patterns through a population-based cohort study. Even after adjustment for confounders, we found a stool pattern (factor 1) that was significantly associated with the prevalence of *pks*^+^ *E. coli*. In addition, this stool pattern was correlated with certain plasma and fecal fatty acids. To the best of our knowledge, this is the first study to show the association of stool patterns with not only the prevalence of *pks*^+^ *E. coli* but also plasma and fecal fatty acids. This association suggests that stool patterns may reflect the presence of tumorigenic bacteria and may provide useful insight about effective strategies for the early discovery of CRC.

Certain risk factors for CRC incidence have been identified in epidemiological studies, including smoking, obesity, diabetes, and high alcohol intake, as well as consumption of red and processed meats [20]. These identified risk factors for CRC [21] and stool status [12–16] have been associated with not only an increased CRC incidence but also potentially adverse gut microbiome profiles. Major research efforts toward the development of stool-based screening tests are ongoing [20], considering that stool status assessment can be conducted through noninvasive self-monitoring without taking time away from routine activities and without the requirement for specialist technology or skill. Recently, the prevalence of *pks*^+^ *E. coli* isolated from the colonic epithelium has been reported to be higher in patients with familial adenomatous polyposis [22], inflammatory bowel disease [23], and CRC [22] than in healthy individuals. Thus, it is important to evaluate the association between comprehensive stool status using a validated tool and the prevalence of tumorigenic bacteria in the gut microbiota.

Our results indicated a significant association between the prevalence of *pks*^+^ *E. coli* and stool pattern. Compared to the sham model, animal models mimicking the natural transmission of colibactin-producing *E. coli* from mothers to neonates have shown lower rates of Firmicutes taxa, Proteobacteria taxa, and microbial species richness, as well as higher DNA repair function [24]. These models have also illustrated an association with gut homeostasis activities, including renewal of the mature epithelium and occurrence of crypt fission [25]. Stool status variables such as shape [12, 13], frequency [12, 15], and color [16] have been associated with higher microbial species richness profiles from fecal analysis in healthy individuals and patients with acute gastroenteritis. Our results support these findings. A previous study showed that the majority of CRC deaths were attributed to non-screening in the United States [26]. Although a causal relationship between the prevalence of *pks*^+^ *E. coli* as a tumorigenic bacteria and an increased risk of CRC has not been established well, it may be potential benefits of evaluating the presence of *pks*^+^ *E. coli* as a target for early prognostication in populations with a high risk of CRC. Our results suggest that stool patterns might be markers associated with the prevalence of tumorigenic bacteria in healthy individuals. Longitudinal objective monitoring of a person’s stool status from serial samples obtained from an individual’s excreta at home, as previously suggested [27], may be the most reasonable and cost-effective method for the early detection of risk factors for CRC.

The stool pattern associated with the presence of *pks*^*+*^ *E. coli* (i.e., factor 1) exhibited a significant negative correlation with the consumption of noodles, alcoholic and non-alcoholic beverages, fruit and vegetable juices, green tea, niacin, and sodium (Supplementary Tables 4 and 5). We had previously shown that green tea consumption was negatively associated with the presence of *pks*^*+*^ *E. coli* [17]., and this previous finding supports our present results. A previous study using fecal samples collected from healthy adults reported that green tea consumption reduces the microbial functional pathways’ abundance relevance to carcinogenesis after 2 weeks of intervention [28]. These results suggest that green tea consumption significantly reduces the prevalence of *pks*^+^ *E. coli.* Via the suppression of the growth of certain microorganisms in the gut microbiome. Taking information from previous studies, we speculate that gut microbiota and dietary components interacted to generate biologically active molecules, which influenced gut secretion and motility, and that this could play a fundamental role in stool status [29, 30] and affect the prevalence of *pks*^+^ *E. coli*. Nonetheless, our results did not substantially change even after adjustment for green tea consumption, indicating that its effect might be weak. Hence, a well-designed study that further evaluates dietary intake, microbiota, and biologically active molecules in the gut in detail is required.

Nutrients derived from ingested food are utilized by the gut microbiome, with certain preferred energy sources such as short-chain fatty acids (SCFAs) for colonocytes [31, 32]. These metabolites can suppress inflammation and carcinogenesis via their effects on immunity, gene expression, and epigenetic modulation [29–32]. Some plasma fatty acids have been shown to be inversely or positively associated with the presence of colon adenomas [33] and an increased risk of CRC in middle-aged adults [34]. Furthermore, studies involving CRC patients have reported lower propionate and butyrate levels [35] and higher valeric acid, isobutyric acid, and isovaleric acid levels [36] in SCFAs derived from fecal samples, as compared to healthy controls. SCFA production is reduced in patients with diarrhea, as compared to those without diarrhea [37]. Additionally, inhibition of SCFA synthesis via the administration of polyethylglycol and antibiotics results in diarrhea [38]. The distal colon transit, as reflected in stool frequency, is associated with not only plasma acetate and fecal SCFAs [39] but also microbiota diversity, especially the Firmicutes taxa (*Faecalibacterium*, *Lactococcus*, and *Roseburia*) [40]. Previous studies reported that certain plasma fatty acids, including α-linoleic acid [41] and certain fecal SCFAs such as propionate [35] and isovaleric acid [36], were associated with a higher CRC incidence. Our results indicated that the stool pattern showing a relationship with the prevalence of *pks*^+^ *E. coli* was also significantly correlated these fatty acids; thus, the findings of these previous studies support our results. While detailed mechanisms and causal relationships should be clarified in further studies, we can conclude that fecal matter is not just a simple waste material but may be useful in the evaluation of the presence of tumorigenic bacteria and fecal fatty acids by stool status via comprehensive examination of variables such as color, shape, frequency, volume, and odor.

The strength of this study is in finding a verified association between stool patterns and plasma and fecal fatty acids. The multifaceted, self-reported questionnaires used for stool status assessment had previously been validated against objective fecal characteristics as well [42]. In addition, we showed that twice self-reported stool status was highly reproducible and believe that it is unlikely for there to have been misclassification when done in this manner. Thus, this study might generate a new hypothesis for the association between the prevalence of *pks*^+^ *E. coli* as tumorigenic bacteria and stool pattern. However, this study has a number of methodological limitations. First, even if the effects of confounders was minimized using multivariate analysis with adjustment for known covariates, being a cross-sectional study, the present study is unable to theorize about the temporal and direct causality of the observed association between stool patterns and the prevalence of *pks*^+^ *E. coli*. Second, this study detected the *clb* gene cluster in the DNA extracted from fecal samples, not in the DNA isolated from the colonic epithelium. A previous study evaluated the prevalence of *pks*^+^ *E. coli* using the selective cultivation method [23]. Our results indicated that the *clb* genes (i.e., *clbA*, *clbJ*, and *clbQ*) were detected as PCR products at a concentration of more than 10 ng/mL of the DNA template. The prevalence of *pks*^+^ *E. coli* might be underestimated because it is considered that the *clb* genes could not be detected at concentrations lower than 10 ng/mL in the fecal samples. However, our previous study [17] showed that the prevalence of *pks*^+^ *E. coli* isolated from fecal matter was relatively similar to that in previous reports investigating the prevalence of *pks*^+^ *E. coli* using the selective cultivation method [23]. Therefore, evaluating the concordance rate of the prevalence of *pks*^+^ *E. coli*, defined using these two different methods for the same subject, will be necessary. Third, although our results showed that a softer stool shape was negatively associated with the prevalence of *pks*^+^ *E. coli*, it is unclear whether the prevalence of *pks*^+^ *E. coli* was lower in participants with diarrhea who had softer stools or whether the reverse was true. In addition, we were unable to completely exclude systematic error due to self-reporting, and we could not account for unmeasured confounding factors associated with stool status in this observational study. For example, stool color is mainly characterized by stercobilin (urobilin), an orange pigment and an oxidized metabolite of urobilinogen [43]. Stercobilin derived from bile pigment is responsible for the brown color of human feces. As we did not directly measure stercobilin and bile acids in all participants, we could not account for their possible effects on the results; nonetheless, our results were similar after adjustment for bile acids in a subsection of participants with available bile acid data. It is necessary to further verify our results with further studies including patients and community-dwelling residents with symptoms such as diarrhea and constipation. Finally, there is the possibility of sampling bias due to the more health-aware nature of the participants in this study than in the general population. Of 750 participants in the Nutrition and Exercise Intervention Study (NEXIS) cohort study, 259 adults agreed to participate. As the participation rate was relatively low, selection bias might have occurred. Additionally, the participants were all living in the Tokyo metropolitan area in Japan and the mean age was 58 years. These limitations may prevent the generalization of our results. Therefore, prospective cohort studies with larger randomized samples should be conducted to further investigate the association between the prevalence of *pks*^+^ *E. coli* and stool patterns.

## Conclusion

These results suggest that an adverse stool pattern is positively associated with the prevalence of *pks*^+^ *E. coli.* Given the rapidly increasing incidence and mortality rates of CRC worldwide, its early discovery is important to both enable people to stay healthy and limit the burden on healthcare-related costs. Therefore, stool patterns may be useful in the evaluation of the presence of tumorigenic bacteria and fecal fatty acids through self-monitoring of stool status without the requirement for specialist technology or skill. Furthermore, it may provide valuable insight about effective strategies for the early discovery of CRC.

## Methods

### Participants and study procedure

This cross-sectional study utilized data from the NEXIS cohort study [17]. This cohort study has been managed by the National Institutes of Biomedical Innovation, Health and Nutrition (NIBIOHN) since 2012 and aims to evaluate the association between gut microbiota and lifestyle, including dietary intake and physical activity (NIBIOHN: no. kenei 102; clinical trial registration number: NCT00926744). Of 750 participants in the NEXIS cohort study, 259 adults aged 27–79 years who were living in the Tokyo metropolitan area in Japan agreed to participate in this study (NIBIOHN: no. kenei 3-04; clinical trial registration number: UMIN000023270). This study was approved by the ethics review board of the Research Ethical Review Committee of the NIBIOHN. After the study procedures and risks associated with the participation in this study were explained, written informed consent was obtained from all participants before data acquisition. This study was conducted in accordance with the principles of the Declaration of Helsinki.

A kit for fecal collection and storage and the questionnaire for the lifestyle survey were mailed to the participants. They were instructed to complete the questionnaire to record pertinent lifestyle variables (e.g. medical history, smoking habit, dietary intake, and stool status) and to collect fecal samples approximately 7 mm in diameter (soybean size) at home. Dietary intake was evaluated using a previously validated brief-type self-administered diet history questionnaire [44]. To measure daily step counts as an objective form of physical activity, we used a triaxial accelerometer (Actimarker EW4800; Panasonic Co., Ltd., Japan). The participants were instructed to bring their fecal samples and questionnaires to the NIBIOHN within a week after answering the questionnaires and finishing the serial fecal collection. Subsequently, they underwent physical and health examinations such as anthropometry and blood tests in the NIBIOHN. Investigators, registered dieticians, or nurses checked the questionnaires and interviewed those with unclear responses or unanswered questions to confirm answers. Blood samples were used as a biochemical examination for conventional risk factors for lifestyle-related diseases, with close attention placed on variables such as low-density lipoprotein-cholesterol, hemoglobin A1c, and triglycerides. The collected feces, serum, and plasma were immediately placed in a sealed container and stored as individual sample types to avoid cross-contamination between samples in a − 20 °C freezer.

Of the participants initially included in this study (*n* = 259), we excluded those with diabetes mellitus (*n* = 13), history of cancer (*n* = 12), cardiovascular disease (*n* = 6), gastrointestinal disease (*n* = 3), and renal failure (*n* = 1). Ultimately, 224 participants were included in the final analysis.

### Confirmation of *pks*^+^*E. coli* by PCR

Bacterial genomic DNA was extracted from frozen fecal samples. Details of this protocol have been reported elsewhere [17, 45, 46]. To confirm the presence of *pks*^+^ *E. coli*, we performed PCR to amplify the genes from the *clb* cluster using bacterial genomic DNA as a template at a concentration of approximately 10 μg/mL. PCR was conducted using SapphireAmp Fast PCR Master Mix (Takara Bio Inc., Shiga, Japan) according to the manufacturer’s protocol. The PCR conditions were as follows: (1) 94 °C for 2 min; (2) 98 °C for 5 s; (3) 63 °C for 5 s; (4) 72 °C for 20 s; (5) repeat (2) to (4) for 30 cycles. The primers used in the PCR experiments were as follows: *clbB* forward primer: 5′-tgttccgttttgtgtggtttcagcg-3′, reverse primer: 5′-gtgcgctgaccattgaagatttccg-3′; *clbJ* forward primer: 5′-tggcctgtattgaaagagcaccgtt-3′, reverse primer: 5′-aatgggaacggttgatgacgatgct-3′; *clbQ* forward primer: 5′-ctgtgtcttacgatggtggatgccg-3′, reverse primer: 5′-gcattaccagattgtcagcatcgcc-3′. The *clb* genes and the expected sizes of their amplicons in PCR were as follows: *clbA*, 613 bp; *clbJ*, 544 bp; and *clbQ*, 430 bp. The amplified DNA was electrophoresed on 3% agarose gel (100 V, 5 min) using the Tris-acetate-EDTA buffer and visualized by ethidium bromide staining. In this analysis, samples amplified with the appropriate amplicon length in the three *clb* genes were defined as *pks*^+^ *E. coli*-positive individuals [17]. Furthermore, in order to determine the minimum detection level of *clb* genes using the PCR method, we verified the genomic DNA purified from a colibactin-producing *E.coli*-50 at each concentration of 0.01–100 ng/mL using a PCR template [45].

### Evaluation of stool status

Stool status was assessed using the multifaced self-reported questionnaire called the “intestinal visible sheet”, which covers the 5-stool status variables (frequency, volume, color, shape, and odor) and was previously developed and validated against objective measurements of fecal characteristics including fecal weight, moisture, hardness, and color in adults [42]. In the NEXIS cohort study, all participants were given similar stool questionnaires assessing both “habitual stool status” and “stool status when collecting fecal samples (excluding stool frequency)” at the same time. We evaluated the reproducibility of the results by comparing these variables because variables evaluated based on self-reporting questionnaires may be affected by recall bias. We used the habitual stool status data in all analysis because it simultaneously evaluated all five stool statuses.

### Measurement of plasma fatty acids

To investigate plasma and fecal fatty acids, we used the frozen stored plasma and fecal samples. Total lipids were extracted from 0.4 mL of plasma following the methodology reported by Folch et al. [47]. After hydrolysis with 5% KOH, fatty acids were extracted with hexane and tricosanoic acid (C23:0) as the internal standard. Methyl-esterified fatty acids were prepared with a trimethylsilylating reagent and subjected to gas chromatography (GC). GC-*electrospray* ionization *mass spectrometry* (ESI/MS) analysis was performed using Shimadzu GC-2010 (Shimadzu Corporation, Kyoto, Japan) equipped with a hydrogen flame ionization detector. A glass column (40 m × 0.3 mm in volume) was coated with diethylene glycol succinate. An AOC-20i autoinjector (Shimadzu Corporation, Kyoto, Japan) was employed for sample injection. Nitrogen gas was used as a carrier gas and delivered at a flow rate of 25 mL min-1. Injection volume was set at 20 μL with a split ratio of 80:1. Column temperature was maintained at 180 °C, and the injection port and detector cell were kept at 350 °C and 250 °C, respectively. The 24 types of plasma fatty acids with chain lengths comprising 12–24 carbons were measured through this analysis.

### Measurement of fecal short-chain fatty acids

Furthermore, 5–10 mg of feces was mixed with 90 μL of Milli-Q and 10 μL of 2 mM internal standard containing acetic acid, butyric acid, and crotonic acid for 5 min. The mixture was homogenized with 50 μL of 36% HCl and 200 μL of 97% diethyl ether and was centrifuged at 3000 rpm for 10 min at room temperature. Subsequently, 80 μL of the supernatant organic layer was transferred to a new glass vial and combined with 16 μL of N-tert-butyldimethylsilyl-N-methyltrifluoroacetamide as a derivatization reagent. The vials were immediately capped tightly with electronic crimper (Agilent), incubated for 20 min in an 80 °C water bath, and then left at room temperature in the dark for 48 h for derivatization. The derivatized samples were analyzed using a GC-MS-TQ8040 gas chromatograph mass spectrometer (Shimadzu Corporation, Kyoto, Japan), and the injection was performed using an AOC-20i autoinjector (Shimadzu Corporation, Kyoto, Japan). The capillary column was a BPX5 column (0.25 mm × 30 m × 0.25 μm; Shimadzu GLC). Pure helium gas was used as a carrier gas and delivered at a flow rate of 1.2 mL min-1. The head pressure was 72.8 kPa with split (split ratio of 30:1). The injection port and interface temperatures were 230 °C and 260 °C, respectively. This analysis measured the 10 types of fecal SCFAs (C1:0–C6:0).

### Statistical analysis

Characteristics of participants with and without *pks*^+^ *E. coli* were compared using the baseline characteristics used in a previous study [17]. Continuous variables were presented as means and standard deviations, with differences between the two groups evaluated using the unpaired t-test. Categorical variables were expressed as numbers and percentages, with differences between the two groups evaluated using the chi-square test.

The agreement, adjacent agreement, and disagreement from the twice-evaluated stool status variables were expressed as numbers and percentages. Disagreement was defined as a difference of more than three categories between each variable. In order to evaluate the reproducibility of the variables in self-reported stool status, we used a weighted κ statistic with 95% CI [48].

To identify the primary stool patterns, we used factorial analysis with varimax rotation (orthogonal transformation) to derive non-correlated factors [49]. This approach maintained a greater interpretability because each factor could be noted independent of the others with distribution explained by the variance among the individual components. We considered the scree plot and eigenvalues to determine the number of factors to retain by minimizing the number of indicators that had high loading on one factor [49]. For these reasons, we identified three stool patterns of interest from the five stool statuses. We considered stable factor load to be scored greater than 0.4 [50]. For every participant, we calculated factor scores for each of the three retained factors by summing the scores multiplied by factor load across all stool statuses. Participants were classified into tertiles (Ts) of factor score for each stool pattern.

The prevalence of *pks*^+^ *E. coli* in each tertile for stool patterns was shown as number of cases and percentage. To adjust for confounders between the prevalence of *pks*^+^ *E. coli* and the stool patterns, we used the likelihood ratio test for multivariate logistic analysis including baseline covariates. The odds ratio (ORs) and 95% CI were estimated. These analyses were verified on two models as follows: Model 1 was adjusted for age (continuous) and sex (female or male); Model 2 was as Model 1 plus mutual adjustment by body mass index (continuous), family history of cancer (yes or no), smoking status (never smoker, past smoker, or current smoker), step count (continuous), alcohol drinker (yes or no), and green tea consumption (continuous). These variables were decided in accordance with covariates used in our previous study [17]. We calculated the prevalence OR of *pks*^+^ *E. coli* with the lowest tertile of each stool pattern as reference. Plasma and fecal fatty acid measurements were expressed as median and interquartile values. Instances wherein fatty acids could not be detected during GC/MS analysis were handled as missing data. In order to evaluate the association of plasma and fecal fatty acids as well as dietary intake with stool patterns, we used Spearman’s correlation coefficient.

For statistical significance, the *p* value was set to < 0.05 (double-sided). All statistical analyses were performed using JMP Pro for Windows (SAS Institute, Inc., Cary, NC, USA).

## Supplementary Information

**Additional file 1: Table S1.** Odds ratios for stool status and prevalence of pks + *E. coli* carrier calculated by multivariate logistic regression analysis. **Table S2.** Correlation between stool status and plasma and fecal fatty acids. **Table S3.** Correlation between fecal short-chain fatty acids and plasma fatty acids. **Table S4.** Correlation between stool patterns and food and beverage consumption. **Table S5.** Correlation between stool patterns and nutrients intake

## Data Availability

The datasets used during the current study are available from the corresponding author on reasonable request.

## Abbreviations

ALT: Alanine transaminase
AST: Aspartate transaminase
CI: Confidence Interval
*clb*: Colibactin
CRC: Colorectal cancer
ESI/MS: Electrospray Ionization Mass Spectrometry
FH: Family History
FPG: Fasting Plasma Glucose
FSI: Fasting Serum Insulin
GC: Gas Chromatography
GTC: Green Tea Consumption
HbA1c: Hemoglobin A1c
HDL-C: High-Density Lipoprotein-Cholesterol
LDL-C: Low-Density Lipoprotein-Cholesterol
NEXIS: Nutrition and Exercise Intervention Study
NIBIOHN: National Institutes of Biomedical Innovation, Health and Nutrition
OR: Odds Ratio
PCR: Polymerase Chain Reaction
*pks*^+^*E. coli*: *Escherichia coli* containing polyketide synthase
Ref: Reference
SCFAs: Short-Chain Fatty Acids
SD: Standard Deviation
Ts: Tertiles
γ-GTP: γ-Glutamyl transpeptidase
