# Supplementary material for: Stool pattern is associated with not only the prevalence of tumorigenic bacteria isolated from fecal matter but also plasma and fecal fatty acids in healthy Japanese adults
Source: BMC Microbiol. 2021 Jun 28;21:196. doi: 10.1186/s12866-021-02255-6 (PMC8240356; doi:10.1186/s12866-021-02255-6)
Supplement: Supplementary file 1 — Additional file 1: Table S1. Odds ratios for stool status and prevalence of pks + E. coli carrier calculated by multivariate logistic regression analysis. Table S2. Correlation between stool status and plasma and fecal fatty acids. Table S3. Correlation between fecal short-chain fatty acids and plasma fatty acids. Table S4. Correlation between stool patterns and food and beverage consumption. Table S5. Correlation between stool patterns and nutrients intake [file 12866_2021_2255_MOESM1_ESM.docx]

**Supporting information**

“Stool pattern is associated with not only the prevalence of tumorigenic bacteria isolated from fecal matter but also plasma and fecal fatty acids in healthy Japanese adults”

Authors: Daiki Watanabe, Haruka Murakami, Harumi Ohno, Kumpei Tanisawa, Kana Konishi, Kikue Todoroki-Mori, Yuta Tsunematsu, Michio Sato, Yuji Ogata, Noriyuki Miyoshi, Naoto Kubota, Jun Kunisawa, Keiji Wakabayashi, Tetsuya Kubota, Kenji Watanabe, and Motohiko Miyachi

**SUPPLEMENTARY TABLES**

**Table S1** Odds ratios for stool status and prevalence of pks+ E. coli carrier calculated by multivariate logistic regression analysis

**Table S2** Correlation between stool status and plasma and fecal fatty acids

**Table S3** Correlation between fecal short-chain fatty acids and plasma fatty acids

**Table S4** Correlation between stool patterns and food and beverage consumption

**Table S5** Correlation between stool patterns and nutrients intake

**Supplementary Methods**

The stool frequency was classified into six categories: (1) 2 or less times per week, (2) 3 times per week, (3) 4 times per week, (4) 5 times per week, (5) 6 times per week, and (6) 7 or more times per week. The stool volume was classified into eight categories: (1) 0.5 unit, (2) 1 unit, (3) 1.5 unit, (4) 2.0 unit, (5) 2.5 unit, (6) 3.0 unit, (7) 3.5 unit, and (8) 4 or more unit. A unit is considerable 2 cm in diameter, 10 cm in length, cylindrical in shape. The stool color was classified into six categories: (1) yellow, (2) light yellowish-brown, (3) yellowish-brown, (4) brown, (5) greenish-dark brown, and (6) dark brown. This was self-evaluated by referring to the stool color illustrated on the handout. The stool shape was classified into seven categories: (1) separate hard lumps (Type 1), (2) lumpy and sausage like (Type 2), (3) a sausage shape with cracks in the surface (Type 3), (4) like a smooth, soft sausage or snake brown (Type 4), (5) soft blobs with clear-cut edges (Type 5), (6) mushy consistency with ragged edges (Type 6), and (7) liquid consistency with no solid pieces (Type 7). The stool odor was classified into four categories: (1) odorless, (2) slight, (3) strong, and (4) very strong.

Because of sparse data, multivariate logistic analysis using stool status was categorized into three groups: frequency, ≥7 times/week (*n* = 139); 5-6 times/week (*n* = 57); ≤4 times/week (*n* = 28): volume, ≤1.5 unit (*n* = 59); 2 unit (*n* = 70); ≥2.5 unit (*n* = 95): color, ≤yellowish-brown (*n* = 83); brown (*n* = 101); ≥dark olive brown (*n* = 40): shape, ≤Type 3 (*n* = 34); Type 4 (*n* = 168); ≥Type 5 (*n* = 22): odor, odorless (*n* = 34); slight (*n* = 173); strong (*n* = 17). We calculated the prevalence OR of the *pks*^+^ *E. coli* due to stool status, based on the frequency (≥7 times/week), volume (≤1.5 unit), color (≤yellowish-brown), shape (≤ Type 3), and odor (odorless) as reference.

**Supplementary Results**

The number of people in each category of stool frequency, volume, color, shape, and odor were as follows: 7 or more times per week (*n* = 139), 6 times per week (*n* = 38), 5 times per week (*n* = 19), 4 times per week (*n* = 9), 3 times per week (*n* = 17), and 2 or less times per week (*n* = 2) in stool frequency; 0.5 unit (*n* = 3), 1 unit (*n* = 20), 1.5 unit (*n* = 36), 2.0 unit (*n* = 70), 2.5 unit (*n* = 29), 3.0 unit (*n* = 39), 3.5 unit (*n* = 12), and 4 or more unit (*n* = 15) in stool volume; yellow (*n* = 0), light yellowish-brown (*n* = 10), yellowish-brown (*n* = 73), brown (*n* = 101), greenish-dark brown (*n* = 34), and dark brown (*n* = 6) in stool color; Type 1 (*n* = 2), Type 2 (*n* = 6), Type 3 (*n* = 26), Type 4 (*n* = 168), Type 5 (*n* = 14), Type 6 (*n* = 8), Type 7 (*n* = 0) in stool shape; odorless (*n* = 34), slight (*n* = 173), strong (*n* = 17), and very strong (*n* = 0) in stool odor.

We evaluated the relationship between the prevalence of *pks*^+^ *E. coli* and stool status by multivariate logistic analysis (Supplementary Table 1). Even after adjusting for covariates by multivariate analysis models, we showed a positive relationship between prevalence of *pks*^+^ *E. coli* and dark stool color [≤Yellowish-brown: reference; Brown: OR, 1.52 (95% CI: 0.77 to 3.00); ≥Dark olive brown: OR, 2.70 (95% CI: 1.17 to 6.23), *p* for trend = 0.020]. In addition, participants with soft stool shape tended to have lower OR for prevalence of *pks*^+^ *E. coli* compared with those who hard stool shape [≤Type 3: reference; Type 4: OR, 0.53 (95% CI: 0.24 to 1.15); ≥Type 5: OR, 0.34 (95% CI: 0.10 to 1.11), *p* for trend = 0.053]. However, there was no association between other stool statuses and the prevalence of *pks*^+^ *E. coli.*

Supplementary Table 2 shows the association of stool status with fatty acid derived from plasma as well as fecal samples. The participants with higher stool frequency were significantly positively correlated with fecal propionate and succinate and were significantly negatively correlated with fecal isobutyrate, isovalenate, and hexanoate. The participants with darker stool color were significantly negatively correlated with plasma erucic acid and α-linoleic acid. The participants with softer stool shape were significantly positively correlated with plasma oleic acid, total n-9 fatty acids, α-linoleic acid, mono fatty acids, and fecal propionate, butyrate, and succinate and were significantly negatively correlated with fecal isobutyrate, isovalenate, and hexanoate. The participants with stronger stool odor were significantly negatively correlated with plasma nervonic acid, eicosapentaenoic acid, docosahexaenoic acid, total n-3 fatty acids, and n-3/n-6 fatty acids. Furthermore, plasma fatty acids showed a significant correlation with fecal short-chain fatty acids (Supplementary Table 3).

The association of stool patterns with dietary intake is shown in Supplementary Tables 4 and 5. The factor 1 score was significantly negatively correlated with the consumption of noodles, alcoholic and non-alcoholic beverages, fruit and vegetable juices, green tea, niacin, and sodium. The factor 2 score was significantly positively correlated with the consumption of foods such as oil, total vegetables, green and yellow vegetables, other vegetables, coffee, and meat and was significantly negatively correlated with the consumption of confectioneries. The factor 3 score was significantly negatively correlated with the consumption of fruits.

**Supplementary Table 1** Odds ratios for stool status and prevalence of *pks*^+^ *E. coli* carrier calculated by multivariate logistic regression analysis

| Stool status | Number or OR (95% CI) | | | | | |  | 1 point change | | *p* for trend ^a^ |
| --- | --- | --- | --- | --- | --- | --- | --- | --- | --- | --- |
| **Frequency (times/week)** | ≥7 | | 5-6 | | ≤4 | |  |  |  |  |
| *n* | 139 | | 57 | | 28 | |  |  |  |  |
| Case [n (%)] | 41 | (25.5) | 20 | (30.3) | 12 | (37.5) |  |  |  |  |
| Model 1 ^b^ | 1.00 | (Ref) | 1.56 | (0.80 to 3.06) | 1.97 | (0.86 to 4.49) |  | 1.43 | (0.97 to 2.11) | 0.070 |
| Model 2 ^c^ | 1.00 | (Ref) | 1.40 | (0.69 to 2.83) | 1.65 | (0.71 to 3.86) |  | 1.30 | (0.87 to 1.95) | 0.199 |
| **Volume (unit)** | ≤1.5 | | 2 | | ≥2.5 | |  |  |  |  |
| *n* | 59 | | 70 | | 95 | |  |  |  |  |
| Case [n (%)] | 14 | (23.7) | 15 | (21.4) | 31 | (32.6) |  |  |  |  |
| Model 1 ^b^ | 1.00 | (Ref) | 0.68 | (0.31 to 1.47) | 1.10 | (0.55 to 2.22) |  | 1.10 | (0.77 to 1.57) | 0.605 |
| Model 2 ^c^ | 1.00 | (Ref) | 0.62 | (0.28 to 1.41) | 1.18 | (0.56 to 2.48) |  | 1.15 | (0.79 to 1.68) | 0.455 |
| **Color** | ≤Yellowish-brown | | Brown | | ≥Dark olive brown | |  |  |  |  |
| *n* | 83 | | 101 | | 40 | |  |  |  |  |
| Case [n (%)] | 17 | (20.5) | 28 | (27.7) | 15 | (37.5) |  | 15 | (37.5) |  |
| Model 1 ^b^ | 1.00 | (Ref) | 1.59 | (0.83 to 3.05) | 2.70 | (1.22 to 6.00) |  | 1.64 | (1.10 to 2.44) | **0.014** |
| Model 2 ^c^ | 1.00 | (Ref) | 1.52 | (0.77 to 3.00) | 2.70 | (1.17 to 6.23) |  | 1.63 | (1.08 to 2.48) | **0.020** |
| **Shape** | ≤Type 3 | | Type 4 | | ≥Type 5 | |  |  |  |  |
| *n* | 34 | | 168 | | 22 | |  |  |  |  |
| Case [n (%)] | 13 | (38.2) | 28 | (25.6) | 15 | (18.2) |  |  |  |  |
| Model 1 ^b^ | 1.00 | (Ref) | 0.54 | (0.26 to 1.13) | 0.31 | (0.10 to 0.98) |  | 0.55 | (0.32 to 0.96) | **0.034** |
| Model 2 ^c^ | 1.00 | (Ref) | 0.53 | (0.24 to 1.15) | 0.34 | (0.10 to 1.11) |  | 0.57 | (0.32 to 1.01) | 0.053 |
| **Odor** | Odorless | | Slight | | Strong | |  |  |  |  |
| *n* | 34 | | 173 | | 17 | |  |  |  |  |
| Case [n (%)] | 7 | (20.6) | 28 | (27.8) | 15 | (29.4) |  |  |  |  |
| Model 1 ^b^ | 1.00 | (Ref) | 0.94 | (0.42 to 2.14) | 1.13 | (0.35 to 3.69) |  | 1.05 | (0.58 to 1.89) | 0.881 |
| Model 2 ^c^ | 1.00 | (Ref) | 0.83 | (0.35 to 1.94) | 1.30 | (0.38 to 4.51) |  | 1.08 | (0.57 to 2.03) | 0.816 |

*Ref* reference. The prevalence rates of *pks*^+^ *E. coli* are shown as numbers of people and percentages. The stool status was evaluated by each category as following; stool frequency [1 point (2 or less times per week) to 6 points (7 or more times per week)], stool volume [1 point (smaller) to 8 points (larger)], stool color [1 point (yellow) to 6 points (dark)], stool shape [1 (severe constipation; separate hard lumps) to 7 (severe diarrhea; liquid consistency with no solid pieces)], and stool odor [1 (no smelly) to 4 (severe smelly)].

^a^ Statistical analysis was carried out using the likelihood ratio test for multivariate logistic analysis, and the odds ratio (ORs) and 95% confidence intervals (CI) were estimated. Bold *p* values are statistically signiﬁcant (*p*< 0.05).

^b^ Model 1 was adjusted for age (continuous) and sex (man or woman).

^c^ Model 2 was as model 1 plus mutual adjustment by BMI (continuous variable), family history of cancer (yes or no), smoking status (never smoker, past smoker, and current smoker), step counts (continuous), alcohol drinker (yes or no), and green tea consumption (continuous).

**Supplementary Table 2** Correlation between stool status and plasma and fecal fatty acids

|  | Stool status | | | | | | | | | |
| --- | --- | --- | --- | --- | --- | --- | --- | --- | --- | --- |
|  | Frequency | | Volume | | Color | | Shape | | Odor | |
| **Plasma** |  |  |  |  |  |  |  |  |  |  |
| Lauric acid | -0.08 |  | -0.05 |  | -0.06 |  | -0.01 |  | -0.02 |  |
| Myristic acid | 0.04 |  | 0.04 |  | -0.07 |  | 0.07 |  | -0.07 |  |
| Palmitic acid | 0.03 |  | 0.04 |  | -0.01 |  | 0.10 |  | -0.06 |  |
| Stearic acid | 0.03 |  | 0.03 |  | 0.01 |  | 0.13 |  | -0.08 |  |
| Arachidic acid | 0.04 |  | -0.06 |  | -0.09 |  | 0.08 |  | -0.04 |  |
| Behenic acid | -0.02 |  | -0.03 |  | -0.05 |  | 0.00 |  | 0.02 |  |
| Lignoceric acid | 0.02 |  | 0.00 |  | 0.00 |  | -0.04 |  | -0.04 |  |
| Total saturated fatty acids | 0.04 |  | 0.04 |  | -0.01 |  | 0.10 |  | -0.07 |  |
| Oleic acid | 0.03 |  | 0.10 |  | -0.04 |  | 0.16 | * | -0.01 |  |
| Eicosenoic acid | 0.09 |  | 0.08 |  | -0.07 |  | 0.12 |  | 0.02 |  |
| 5,8,11-Eicosatrienoic acid | 0.01 |  | 0.00 |  | -0.02 |  | 0.03 |  | 0.11 |  |
| Erucic acid | -0.24 |  | 0.01 |  | -0.29 | * | 0.04 |  | 0.07 |  |
| Nervonic acid | 0.08 |  | 0.04 |  | -0.02 |  | 0.02 |  | -0.15 | * |
| Total n-9 fatty acids | 0.05 |  | 0.10 |  | -0.04 |  | 0.16 | * | -0.02 |  |
| Palmitoleic acid | 0.03 |  | 0.02 |  | -0.09 |  | 0.12 |  | -0.08 |  |
| Linoleic acid | -0.02 |  | 0.05 |  | 0.01 |  | 0.06 |  | 0.01 |  |
| γ-linolenic acid | 0.02 |  | -0.03 |  | -0.01 |  | 0.07 |  | 0.07 |  |
| Eicosadienoic acid | -0.01 |  | 0.02 |  | -0.05 |  | 0.12 |  | 0.06 |  |
| Dihomo-gamm-linolenic acid | 0.01 |  | -0.02 |  | -0.06 |  | 0.04 |  | 0.12 |  |
| Arachidonic acid | -0.01 |  | 0.04 |  | 0.06 |  | 0.02 |  | -0.04 |  |
| Docosatetraenoic acid | -0.08 |  | 0.03 |  | 0.04 |  | 0.03 |  | 0.09 |  |
| Total n-6 fatty acids | -0.03 |  | 0.03 |  | 0.02 |  | 0.05 |  | 0.00 |  |
| Myristoleic acid | -0.01 |  | 0.01 |  | -0.12 |  | 0.07 |  | -0.08 |  |
| α-linoleic acid | 0.00 |  | 0.03 |  | -0.13 | * | 0.16 | * | -0.03 |  |
| Eicosapentaenoic acid | 0.12 |  | -0.06 |  | -0.04 |  | 0.10 |  | -0.21 | ** |
| Docosapentaenoic acid | 0.07 |  | 0.02 |  | 0.02 |  | 0.03 |  | -0.11 |  |
| Docosahexaenoic acid | 0.07 |  | 0.02 |  | -0.02 |  | 0.05 |  | -0.16 | * |
| Total n-3 fatty acids | 0.11 |  | 0.02 |  | -0.04 |  | 0.09 |  | -0.19 | ** |
| Mono fatty acids | 0.04 |  | 0.09 |  | -0.05 |  | 0.16 | * | -0.04 |  |
| Polyunsaturated fatty acid | 0.04 |  | 0.04 |  | 0.00 |  | 0.07 |  | -0.10 |  |
| n-3/n-6 fatty acids | 0.12 |  | -0.01 |  | -0.06 |  | 0.06 |  | -0.19 | ** |
| Total fatty acids | 0.04 |  | 0.06 |  | -0.01 |  | 0.11 |  | -0.07 |  |
| **Fecal** |  |  |  |  |  |  |  |  |  |  |
| Formate | -0.07 |  | -0.10 |  | 0.01 |  | -0.18 |  | -0.10 |  |
| Acetate | 0.10 |  | 0.09 |  | -0.04 |  | 0.07 |  | 0.06 |  |
| Propionate | 0.28 | ** | 0.13 |  | -0.10 |  | 0.26 | ** | -0.07 |  |
| Lactate | 0.19 |  | -0.06 |  | 0.23 |  | -0.15 |  | 0.32 |  |
| Isobutyrate | -0.25 | ** | -0.10 |  | -0.03 |  | -0.16 | * | -0.08 |  |
| Butyrate | 0.09 |  | 0.02 |  | 0.00 |  | 0.18 | * | -0.08 |  |
| Succinate | 0.17 | ***** | 0.01 |  | -0.03 |  | 0.25 | ** | 0.03 |  |
| Isovalerate | -0.28 | ** | -0.12 |  | 0.02 |  | -0.20 | ** | -0.08 |  |
| Valerate (Pentanoate) | -0.15 |  | -0.03 |  | 0.00 |  | -0.09 |  | -0.04 |  |
| Hexanoate | -0.32 | ** | -0.05 |  | 0.11 |  | -0.20 | ** | 0.12 |  |
| Total short-chain fatty acids | 0.16 | ***** | 0.09 |  | -0.05 |  | 0.16 | ***** | -0.02 |  |

Statistical analysis was performed by Spearman’s correlation analysis. The statistical significance for *p* value is indicated as follows; if *p* < 0.05 single asterisk (*) or if *p* < 0.01 double asterisk (**). If the results presented a positive correlation, the participants with higher stool frequency, volume, darker, softer, or odor mean to relate higher plasma and fecal fatty acids (conversely, a negative correlation indicates that they mean lower it).

**Supplementary Table 3** Correlation between fecal short-chain fatty acids and plasma fatty acids

| Plasma fatty acid | Structure | Fecal short-chain fatty acids | | | | | | | | | | | | | | | | | | | | | |
| --- | --- | --- | --- | --- | --- | --- | --- | --- | --- | --- | --- | --- | --- | --- | --- | --- | --- | --- | --- | --- | --- | --- | --- |
|  |  | Formate (*n* = 58) | | Acetate (*n* = 161) | | Propionate (*n* = 161) | | Lactate (*n* = 19) | | Isobutyrate (*n* = 161) | | Butyrate (*n* = 161) | | Succinate (*n* = 143) | | Isovalerate (*n* = 161) | | Valerate (*n* = 160) | | Hexanoate (*n* = 161) | | Total (*n* = 161) | |
| Lauric acid | C12:0 | 0.27 | ***** | 0.04 |  | 0.10 |  | -0.24 |  | 0.14 |  | 0.00 |  | -0.11 |  | 0.13 |  | 0.07 |  | 0.16 | ***** | 0.03 |  |
| Myristic acid | C14:0 | 0.28 | ***** | 0.06 |  | 0.19 | ***** | -0.43 |  | 0.07 |  | 0.04 |  | -0.05 |  | 0.06 |  | 0.07 |  | 0.12 |  | 0.08 |  |
| Palmitic acid | C16:0 | 0.15 |  | 0.04 |  | 0.17 | ***** | -0.52 | ***** | 0.07 |  | 0.04 |  | -0.04 |  | 0.08 |  | 0.07 |  | 0.10 |  | 0.08 |  |
| Stearic acid | C18:0 | 0.05 |  | -0.07 |  | 0.04 |  | -0.42 |  | 0.07 |  | 0.00 |  | -0.12 |  | 0.07 |  | 0.05 |  | 0.11 |  | -0.03 |  |
| Arachidic acid | C20:0 | -0.14 |  | -0.09 |  | -0.02 |  | -0.47 | * | 0.11 |  | -0.07 |  | -0.06 |  | 0.14 |  | -0.04 |  | 0.00 |  | -0.09 |  |
| Behenic acid | C22:0 | -0.14 |  | -0.08 |  | -0.12 |  | -0.30 |  | 0.12 |  | -0.08 |  | -0.12 |  | 0.14 |  | -0.07 |  | 0.08 |  | -0.14 |  |
| Lignoceric acid | C24:0 | -0.03 |  | -0.10 |  | -0.15 |  | 0.24 |  | -0.02 |  | -0.08 |  | -0.02 |  | 0.02 |  | -0.10 |  | 0.15 |  | -0.13 |  |
| Total saturated fatty acids |  | 0.15 |  | 0.01 |  | 0.13 |  | -0.48 | * | 0.07 |  | 0.02 |  | -0.06 |  | 0.08 |  | 0.06 |  | 0.11 |  | 0.05 |  |
| Oleic acid | C18:1n-9 | 0.13 |  | 0.08 |  | 0.17 | ***** | -0.57 | * | 0.01 |  | 0.07 |  | 0.00 |  | 0.01 |  | 0.08 |  | 0.07 |  | 0.11 |  |
| Eicosenoic acid | C20:1n-9 | 0.17 |  | -0.05 |  | 0.10 |  | -0.43 |  | -0.09 |  | -0.08 |  | 0.03 |  | -0.07 |  | -0.08 |  | -0.02 |  | 0.00 |  |
| 5,8,11-Eicosatrienoic acid | C20:3n-9 | 0.20 |  | 0.03 |  | 0.06 |  | -0.24 |  | -0.01 |  | 0.05 |  | -0.14 |  | 0.00 |  | 0.03 |  | 0.18 | ***** | 0.04 |  |
| Erucic acid | C22:1n-9 | 0.08 |  | 0.04 |  | 0.09 |  | 0.00 |  | 0.04 |  | -0.04 |  | 0.12 |  | -0.03 |  | 0.14 |  | 0.13 |  | 0.09 |  |
| Nervonic acid | C24:1n-9 | -0.07 |  | -0.13 |  | -0.15 |  | 0.23 |  | -0.05 |  | -0.11 |  | 0.03 |  | -0.01 |  | -0.12 |  | 0.07 |  | -0.13 |  |
| Total n-9 fatty acids |  | 0.12 |  | 0.07 |  | 0.15 |  | -0.55 | * | 0.00 |  | 0.05 |  | 0.00 |  | 0.00 |  | 0.06 |  | 0.08 |  | 0.10 |  |
| Palmitoleic acid | C16:1n-7 | 0.23 |  | 0.12 |  | 0.26 | ** | -0.48 | * | 0.10 |  | 0.11 |  | 0.02 |  | 0.09 |  | 0.15 |  | 0.08 |  | 0.17 | ***** |
| Linoleic acid | C18:2n-6 | -0.02 |  | -0.06 |  | -0.06 |  | -0.47 | * | -0.01 |  | 0.03 |  | -0.02 |  | 0.01 |  | -0.03 |  | 0.04 |  | -0.07 |  |
| γ-linolenic acid | C18:3n-6 | 0.15 |  | 0.00 |  | 0.08 |  | 0.03 |  | 0.06 |  | 0.02 |  | -0.12 |  | 0.07 |  | 0.03 |  | 0.17 | ***** | 0.02 |  |
| Eicosadienoic acid | C20:2n-6 | 0.26 |  | 0.04 |  | 0.04 |  | -0.50 | * | -0.06 |  | 0.06 |  | -0.04 |  | -0.06 |  | -0.03 |  | 0.09 |  | 0.04 |  |
| Dihomo-gamm-linolenic acid | C20:3n-6 | 0.23 |  | 0.05 |  | 0.07 |  | -0.08 |  | 0.01 |  | 0.06 |  | -0.15 |  | 0.00 |  | 0.05 |  | 0.18 | ***** | 0.06 |  |
| Arachidonic acid | C20:4n-6 | -0.07 |  | -0.12 |  | 0.00 |  | -0.19 |  | 0.04 |  | -0.07 |  | -0.10 |  | 0.06 |  | 0.01 |  | 0.16 | ***** | -0.08 |  |
| Docosatetraenoic acid | C22:4n-6 | 0.23 |  | -0.06 |  | 0.02 |  | -0.43 |  | 0.05 |  | -0.03 |  | -0.12 |  | 0.07 |  | 0.05 |  | 0.19 | ***** | -0.03 |  |
| Total n-6 fatty acids |  | -0.02 |  | -0.08 |  | -0.05 |  | -0.50 | * | 0.01 |  | 0.01 |  | -0.06 |  | 0.04 |  | -0.01 |  | 0.09 |  | -0.08 |  |
| Myristoleic acid | C14:1n-5 | 0.21 |  | 0.06 |  | 0.13 |  | -0.48 | * | 0.11 |  | -0.01 |  | 0.00 |  | 0.11 |  | 0.15 |  | 0.08 |  | 0.07 |  |
| α-linoleic acid | C18:3n-3 | 0.19 |  | 0.08 |  | 0.08 |  | -0.43 |  | -0.05 |  | 0.04 |  | 0.07 |  | -0.05 |  | -0.04 |  | 0.00 |  | 0.08 |  |
| Eicosapentaenoic acid | C20:5n-3 | -0.08 |  | 0.05 |  | 0.18 | ***** | -0.08 |  | 0.13 |  | -0.01 |  | -0.06 |  | 0.12 |  | 0.04 |  | 0.02 |  | 0.06 |  |
| Docosapentaenoic acid | C22:5n-3 | 0.04 |  | -0.02 |  | 0.02 |  | 0.06 |  | -0.05 |  | -0.04 |  | -0.01 |  | -0.03 |  | -0.03 |  | 0.15 |  | -0.01 |  |
| Docosahexaenoic acid | C22:6n-3 | -0.03 |  | -0.01 |  | 0.00 |  | 0.22 |  | -0.04 |  | -0.05 |  | 0.01 |  | -0.03 |  | -0.05 |  | 0.11 |  | -0.01 |  |
| Total n-3 fatty acids |  | 0.00 |  | 0.01 |  | 0.05 |  | 0.14 |  | -0.03 |  | -0.04 |  | 0.03 |  | -0.02 |  | -0.06 |  | 0.09 |  | 0.02 |  |
| Mono fatty acids |  | 0.14 |  | 0.06 |  | 0.17 | ***** | -0.52 | * | 0.01 |  | 0.06 |  | 0.01 |  | 0.01 |  | 0.06 |  | 0.08 |  | 0.10 |  |
| Polyunsaturated fatty acid |  | 0.00 |  | -0.07 |  | -0.02 |  | -0.42 |  | 0.00 |  | -0.03 |  | -0.04 |  | 0.03 |  | -0.04 |  | 0.10 |  | -0.06 |  |
| n-3/n-6 fatty acids |  | 0.00 |  | 0.05 |  | 0.07 |  | 0.27 |  | -0.01 |  | -0.02 |  | 0.02 |  | -0.01 |  | -0.03 |  | 0.08 |  | 0.06 |  |
| Total fatty acids |  | 0.09 |  | -0.01 |  | 0.08 |  | -0.47 | * | 0.02 |  | 0.00 |  | -0.04 |  | 0.04 |  | 0.01 |  | 0.10 |  | 0.01 |  |

Statistical analysis was performed by Spearman’s correlation analysis. The statistical significance for *p* value is indicated as follows; if *p* < 0.05 single asterisk (*) or if *p* < 0.01 double asterisk (**).

**Supplementary Table 4** Correlation between stool patterns and food and beverage consumption

| Food and beverages | Unit | Median | Interquartile range | Stool status pattern | | | | | |
| --- | --- | --- | --- | --- | --- | --- | --- | --- | --- |
|  |  |  |  | 1 | | 2 | | 3 | |
| Cereals | g/day | 291.2 | (201.2 to 360.4) | -0.07 |  | 0.13 |  | 0.08 |  |
| Rice | g/day | 208.0 | (117.0 to 260.0) | -0.02 |  | 0.13 |  | 0.06 |  |
| Noodles | g/day | 48.0 | (30.9 to 80.3) | -0.14 | * | 0.02 |  | 0.05 |  |
| Bread | g/day | 35.9 | (20.0 to 56.0) | 0.03 |  | 0.02 |  | 0.05 |  |
| Pulses | g/day | 56.1 | (36.9 to 91.7) | 0.04 |  | 0.04 |  | -0.13 |  |
| Potatoes | g/day | 45.0 | (20.0 to 60.0) | -0.07 |  | 0.12 |  | 0.02 |  |
| Sugar and confectioneries | g/day | 43.3 | (27.2 to 66.3) | -0.01 |  | -0.12 |  | 0.09 |  |
| Sugar | g/day | 3.8 | (2.1 to 5.3) | -0.12 |  | 0.02 |  | 0.04 |  |
| Confectioneries | g/day | 39.3 | (23.6 to 60.7) | 0.01 |  | -0.14 | * | 0.08 |  |
| Oil | g/day | 9.6 | (6.9 to 13.5) | 0.02 |  | 0.19 | ** | 0.13 |  |
| Fruits | g/day | 137.9 | (73.7 to 198.9) | -0.03 |  | 0.04 |  | -0.15 | * |
| Total vegetables | g/day | 271.0 | (202.6 to 386.3) | -0.04 |  | 0.16 | * | -0.01 |  |
| Green and yellow vegetables | g/day | 102.1 | (62.6 to 153.6) | -0.01 |  | 0.13 | * | 0.04 |  |
| Other vegetables | g/day | 133.1 | (99.9 to 202.1) | -0.02 |  | 0.16 | * | -0.05 |  |
| Pickled vegetables | g/day | 8.2 | (2.7 to 18) | -0.13 |  | 0.04 |  | 0.07 |  |
| Mushrooms | g/day | 11.3 | (5.1 to 20.5) | -0.10 |  | 0.08 |  | -0.09 |  |
| Seaweeds | g/day | 12.3 | (4.9 to 19.7) | -0.09 |  | 0.07 |  | -0.05 |  |
| Alcoholic beverages | g/day | 30.3 | (1.8 to 166.1) | -0.14 | * | 0.03 |  | 0.08 |  |
| Non-alcoholic beverages | g/day | 657.7 | (461.7 to 867) | -0.18 | ** | 0.07 |  | 0.06 |  |
| Fruit and vegetable juice | g/day | 15.4 | (0 to 71.4) | -0.14 | * | 0.00 |  | -0.03 |  |
| Green tea | g/day | 150.0 | (53.5 to 375) | -0.16 | * | -0.06 |  | 0.02 |  |
| Black and oolong tea | g/day | 53.6 | (10.0 to 123.7) | 0.00 |  | -0.02 |  | -0.03 |  |
| Coffee | g/day | 375.0 | (107.1 to 375) | -0.07 |  | 0.17 | * | 0.12 |  |
| Soft drinks | g/day | 13.3 | (0 to 32.9) | -0.11 |  | -0.09 |  | 0.03 |  |
| Fish and shellﬁsh | g/day | 72.3 | (48.2 to 112.2) | -0.05 |  | -0.03 |  | 0.00 |  |
| Meat | g/day | 71.2 | (52.6 to 95.9) | -0.08 |  | 0.15 | * | 0.05 |  |
| Eggs | g/day | 32.7 | (23.5 to 54.9) | -0.04 |  | 0.13 |  | 0.03 |  |
| Dairy products | g/day | 160.7 | (106.4 to 212.8) | -0.03 |  | 0.05 |  | -0.07 |  |

Statistical analysis was performed by Spearman’s correlation analysis. The statistical significance for *p* value is indicated as follows; if *p* < 0.05 single asterisk (*) or if *p* < 0.01 double asterisk (**). If the results presented a positive correlation, the participants with higher adherence to each stool patterns mean to relate higher food and beverage consumption (conversely, a negative correlation indicates that they mean lower it).

**Supplementary Table 5** Correlation between stool patterns and nutrients intake

| Food and beverages | Unit | Mean | SD | Stool status pattern | | | | | |
| --- | --- | --- | --- | --- | --- | --- | --- | --- | --- |
|  |  |  |  | 1 | | 2 | | 3 | |
| Energy | kcal/day | 1742 | 498 | -0.10 |  | 0.19 | ** | 0.05 |  |
| Protein | g/day | 72.8 | 24.5 | -0.09 |  | 0.12 |  | 0.02 |  |
| Fat | g/day | 56.2 | 19.0 | -0.05 |  | 0.15 | * | 0.05 |  |
| Saturated fat | g/day | 15.4 | 5.6 | -0.02 |  | 0.13 |  | 0.03 |  |
| Monounsaturated fat | g/day | 19.9 | 7.1 | -0.06 |  | 0.17 | * | 0.06 |  |
| Polyunsaturated fat | g/day | 13.3 | 4.6 | -0.07 |  | 0.16 | * | 0.05 |  |
| n-6 polyunsaturated fat | g/day | 10.4 | 3.6 | -0.07 |  | 0.17 | * | 0.07 |  |
| n-3 polyunsaturated fat | g/day | 2.83 | 1.17 | -0.08 |  | 0.10 |  | 0.03 |  |
| Marine-origin n-3 polyunsaturated fat ^a^ | g/day | 1.08 | 0.67 | -0.08 |  | 0.00 |  | -0.01 |  |
| Eicosapentaenoic acid | mg/day | 366 | 241 | -0.08 |  | -0.01 |  | -0.01 |  |
| Docosahexaenoic acid | mg/day | 608 | 365 | -0.08 |  | 0.00 |  | -0.01 |  |
| α-linolenic acid | mg/day | 1614 | 624 | -0.05 |  | 0.18 | ** | 0.04 |  |
| Cholesterol | mg/day | 411 | 175 | -0.04 |  | 0.10 |  | 0.03 |  |
| Carbohydrate | g/day | 216 | 70 | -0.09 |  | 0.14 | * | 0.08 |  |
| Total dietary ﬁber | g/day | 13.2 | 4.8 | -0.06 |  | 0.16 | * | 0.00 |  |
| Soluble dietary ﬁber | g/day | 3.4 | 1.3 | -0.06 |  | 0.15 | * | 0.00 |  |
| Insoluble dietary ﬁber | g/day | 9.3 | 3.3 | -0.06 |  | 0.17 | * | 0.00 |  |
| Alcohol | g/day | 9.6 | 15.4 | -0.10 |  | 0.03 |  | 0.05 |  |
| Retinol | µg/day | 513 | 420 | -0.05 |  | 0.06 |  | 0.04 |  |
| Vitamin A (retinol equivalent) ^b^ | µg/day | 894 | 492 | -0.05 |  | 0.14 | * | 0.05 |  |
| α-carotene | µg/day | 496 | 341 | -0.01 |  | 0.23 | ** | -0.02 |  |
| β-carotene | µg/day | 4099 | 2449 | 0.02 |  | 0.17 | ** | 0.01 |  |
| β-carotene equivalente ^c^ | µg/day | 4539 | 2642 | 0.02 |  | 0.18 | ** | 0.01 |  |
| Cryptoxanthin | µg/day | 380 | 321 | -0.07 |  | 0.08 |  | -0.04 |  |
| α-tocopherol | mg/day | 8.07 | 2.90 | -0.06 |  | 0.15 | * | 0.03 |  |
| Vitamin K | µg/day | 355 | 173 | -0.02 |  | 0.09 |  | 0.01 |  |
| Thiamin | mg/day | 0.85 | 0.29 | -0.09 |  | 0.19 | ** | 0.01 |  |
| Riboﬂavin | mg/day | 1.45 | 0.47 | -0.12 |  | 0.09 |  | 0.01 |  |
| Niacin | mg/day | 18.9 | 6.6 | -0.13 | * | 0.16 | * | 0.05 |  |
| Vitamin B6 | mg/day | 1.40 | 0.47 | -0.11 |  | 0.18 | ** | 0.00 |  |
| Vitamin B12 | µg/day | 10.56 | 5.79 | -0.06 |  | -0.01 |  | 0.02 |  |
| Folate | µg/day | 389 | 151 | -0.08 |  | 0.13 |  | 0.01 |  |
| Pantothenic acid | mg/day | 6.98 | 2.19 | -0.11 |  | 0.17 | * | 0.01 |  |
| Vitamin C | mg/day | 136 | 62 | -0.08 |  | 0.13 |  | -0.05 |  |
| Sodium | mg/day | 4092 | 1300 | -0.15 | * | 0.12 |  | 0.04 |  |
| Potassium | mg/day | 2908 | 998 | -0.09 |  | 0.18 | ** | 0.01 |  |
| Calcium | mg/day | 624 | 255 | -0.06 |  | 0.06 |  | -0.03 |  |
| Magnesium | mg/day | 275 | 89 | -0.09 |  | 0.16 | * | 0.04 |  |
| Phosphorus | mg/day | 1132 | 390 | -0.08 |  | 0.13 | * | 0.02 |  |
| Iron | mg/day | 8.49 | 2.95 | -0.08 |  | 0.13 | * | 0.03 |  |
| Zinc | mg/day | 8.27 | 2.60 | -0.09 |  | 0.18 | ** | 0.03 |  |
| Copper | mg/day | 1.13 | 0.35 | -0.09 |  | 0.15 | * | 0.02 |  |
| Manganese | mg/day | 3.00 | 1.04 | -0.13 |  | 0.06 |  | 0.03 |  |

Statistical analysis was performed by Spearman’s correlation analysis. The statistical significance for *p* value is indicated as follows; if *p* < 0.05 single asterisk (*) or if *p* < 0.01 double asterisk (**). If the results presented a positive correlation, the participants with higher adherence to each stool patterns mean to relate higher nutrients intake (conversely, a negative correlation indicates that they mean lower it).

^a^ Sum of eicosapentaenoic acid, docosapentaenoic acid, and docosahexaenoic acid.

^b^ Sum of retinol, β-carotene/12, α-carotene/24, and cryptoxanthin/24.

^c^ Sum of β-carotene, α-carotene/2, and cryptoxanthin/2.
